# Insights into garlic (*Allium Sativum*)’s nutrigenomics-associated fly-repellent potency in cattle

**DOI:** 10.1007/s11250-025-04406-7

**Published:** 2025-04-03

**Authors:** Fhulufhelo Mudau, Obioha Durunna, Cletos Mapiye, Farouk Semwogerere, Frans Hagg, Emiliano Raffrenato, Annelin Molotsi

**Affiliations:** 1 Department of Animal Sciences, Faculty of Agrisciences, Stellenbosch University, Matieland, 7602 South Africa; 2 Department of Applied Research, Lakeland College, Vermilion, AB T9X 1K5 Canada; 3 Animal Resources Research Program, Abi Zonal Agricultural Research and Development Institute (Abi ZARDI). National Agricultural Research Organization, P. O. Box 219, Arua, Uganda; 4 Allied Nutrition, Centurion, 0157 South Africa; 5 Department of Agriculture and Animal Health, University of South Africa, PO Box 392, Johannesburg, 0003 South Africa

**Keywords:** Botanical pesticides, Cattle, Garlic supplementation, Gene expression, Fly infestation, Nutrigenomics

## Abstract

Despite effective control of flies using synthetic pesticides, fly resistance and environmental contamination have led to the inadequacy of this strategy. The use of integrated pest management approaches has since been advocated in contemporary research to sustainably control fly populations. Recent studies have found garlic (*Allium Sativum*) and its derivative bioactive compounds to possess insect-repellent attributes among other key health and production enhancing properties. This highlights the potential of garlic as a botanical pesticide to control flies in cattle. Moreover, the ability of cattle to naturally repel flies is influenced by animal genetic predisposition. The dietary garlic supplementation and gene interaction in disease resistance could also be an influential factor in repelling flies in cattle. Transcriptomics has emerged as a valuable tool in animal breeding and genetics which allows identification of trait-associated genes and understanding of complex interactions between dietary nutrients and animal genome expression. This paper explores the nutrigenomic effects of garlic supplementation on cattle and its contribution towards fly repellence efficacy in cattle. It was concluded that garlic supplementation in cattle diets could offer a sustainable approach to managing fly infestations in cattle farming. These findings underscore the importance of further research to validate these assertions and optimise the use of garlic to control flies in cattle under different production systems.

## Introduction

The repercussions of food and nutrition insecurity are manifold, often extending beyond the realms of mere access to food. In regions where livestock rearing is a crucial source of nourishment and economic stability, the growing concern of fly infestation among cattle compounds the challenges (Ilemobade 2009; Saini et al. 2017; Moreki et al. 2022). Flies relentlessly attack and torment cattle inflicting a lot of stress to the animals that results in impaired productivity, hindering producers' ability to meet the demands of meat and dairy products (Taylor et al. 2012; Moreki et al. 2022). Consequently, this exacerbates the strain on already fragile food systems, intensifying food and nutrition insecurity for communities reliant on livestock. Obligate blood-sucking arthropods such as the stable fly (*Stomoxys calcitrans*) and the horn fly (*Haematobia irritans irritans*) can also act as mechanical and biological vectors of diseases, such as lumpy skin disease virus and bovine leukaemia virus (Panei et al. 2019; Sprygin et al. 2019; Makhahlela et al. 2022), respectively. In South Africa, the stable fly (*Stomoxys calcitrans*), the house fly (*Musca domestica*), and a few horse fly species (*Tabanidae*) are the most economically detrimental pests on beef cattle in feedlots (Evert 2014), causing an additional expense of chemical fly control (Evert and Van Hamburg 2014; Makhahlela et al. 2022). For example, the annual economic losses associated with these flies account for over US$ 2 billion in the United States (Taylor et al. 2012).

Filthy flies have over the years been effectively controlled with several chemical pesticides or larvicides; however, reliance on their use has become risky and inadequate due to the insecticidal resistance that has been amply reported (Foil and Hogsette 1994; Cook 2020). This is mostly due to the under- and over-application of insecticides over time, which led to flies developing immunity against currently used insecticides (Iqbal et al. 2014; Siddiqui et al. 2023). Furthermore, these synthetic chemicals’ persistent residues can contaminate food and the environment (Carvalho 2006). Owing to an increase in the incidence of insecticide resistance, and environmental contamination concerns, it is thus imperative to investigate alternative mitigation strategies (Iqbal et al. 2014; Mohammed et al. 2016). The need for synthetic insecticide application to control pest flies can be reduced by employing integrated pest management (IPM) approaches, using biological, cultural, physical/mechanical, and chemical management tools. This IPM approach is one of the effective methods for controlling the incidence of flies in livestock production (Cook 2020; Nisar et al. 2021; Moreki et al. 2022).

In recent years, researchers have grown interest in harnessing the potential of locally available botanicals or phytogenics to combat insect pests (Leesombun et al. 2022; Divekar 2023). Phytogenic compounds extracted from botanical plants such as *Melinis minutiflora* grass, moringa (*Moringa oleifera),* black pepper *(Piper nigrum),* soybean (*Glycine max*), chaste tree seeds (*Vitex agnus castus*), oregano (﻿*Plectranthus amboinicus*), and garlic (*Allium sativum)* have been investigated for their effectiveness in controlling dipteran insect populations, including flies and mosquitoes (Showler 2017; Cook 2020; Leesombun et al. 2022). Exploiting defense mechanism of these plants against pests is one possibly cost-effective and sustainable fly control approach that can complement the existing IPM approach. Garlic supplementation has been a topic of interest in livestock production for its potential to control pests (El-Saber Batiha et al. 2020; Ding et al. 2023; Abd El-Ghany 2024), improve animal health (Banerjee and Maulik 2002; Shokrollahi et al. 2016) and growth performance (Ikyume et al. 2017). Garlic’s repellent and biocidal properties makes it a potential sustainable biopesticide.

Selecting fly-resistant traits in cattle breeding programs, particularly through genetic predisposition and dietary interventions such as garlic supplementation could offer a sustainable solution to mitigate the impacts of fly infestation, safeguarding both animal welfare and food and nutrition security in vulnerable regions. This dual strategy could significantly reduce reliance on synthetic pesticides, lower production costs, and minimise environmental impact. Literature indicates that fly resistance is a heritable trait, with heritability estimates around 0.25 (Basiel et al. 2021), suggesting that selective breeding can effectively enhance this trait in cattle populations. Cattle producers can identify and select individuals that exhibit natural resistance to flies, resulting in herds that are less affected by pest flies over time. Integrating garlic into cattle diets offers additional benefits. Garlic possesses natural pesticidal properties and may enhance the expression of genes related to immunity and stress tolerance through nutrigenomic interactions (Charron et al. 2016). This dietary approach can complement genetic resistance strategies, potentially leading to cattle that not only resist fly infestations but also exhibit improved feed efficiency (FE) and growth. By focusing on the interactions between diet and genetic predispositions, researchers can develop targeted strategies that maximise the synergistic effects of both genetic resistance and dietary enhancements. The current scoping review explores the intricate mechanisms underpinning garlic supplementation in livestock production, specifically focusing on its efficacy in repelling ectoparasites, nutrigenomic effects on gene expression, modulating immune responses, and impacting metabolic pathways. Identifying key gene-diet interactions could lead to the development of cattle breeds that are not only naturally resistant to flies but also more resilient in various environmental conditions. By doing so, the review sets the stage for future research aimed at refining these strategies and validating their effectiveness in diverse livestock production systems.

## Review methodology

**Fig. 1:**
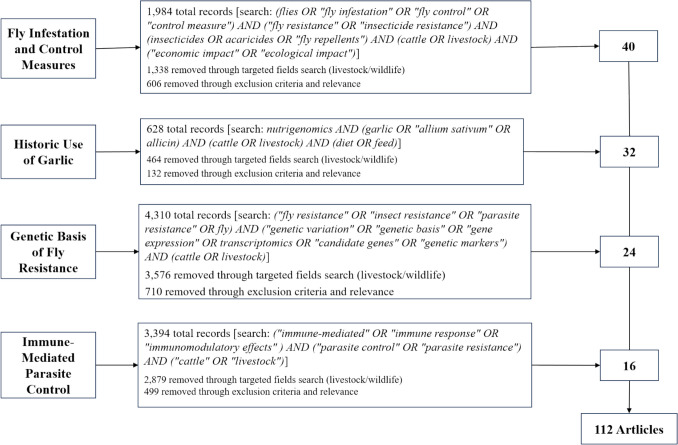
Scoping search strategy and literature selection

A scoping search of peer-reviewed literature across multiple academic and grey literature databases, including PubMed, Scopus, Web of Science, ScienceDirect, SpringerLink, Google Scholar, Google, and Core, with a particular focus on fly abundance in cattle, control measures, and the impact of garlic supplementation, focusing on nutrigenomics and the genetic basis of fly resistance in cattle. This was accomplished using broad Boolean search strings, as indicated in Fig. 1. Where necessary, ResearchGate was used to access pre-print articles. Table 1 provides more information about the inclusion and exclusion criteria of the reviewed literature.

**Table 1:** Inclusion and exclusion criteria of the reviewed literature

Criteria	Inclusion	Exclusion
Focus	Studies or reports on themes on fly abundance in cattle, control measures, and the impact of garlic supplementation, focusing on nutrigenomics and the genetic basis of fly resistance in cattle	
Search	﻿Restricted to peer-reviewed papers from established journals and papers/reports published within an organization (ex. pre-print, etc.)	﻿Non-peer-reviewed; non-reputable organization-related reports and predatory journals
Language	English	Non-English
Publication year	Eligible paper from all years	None
Context	Globally, with a specific interest in South Africa for certain sections	None
Fly infestation	Livestock (ruminants, particularly cattle) and wildlife	Plants and fruits

## Fly infestation in cattle and control measures

### Key filth flies, distributions, and their implications in Cattle Production

The global population of known fly species is estimated to be approximately 160,000, and they are characterized by having only two wings and belonging to the order Diptera, which translates to "two wings" in Latin. In the Afrotropical Region, a total of 108 families of Diptera are present, accounting for approximately 38% of the 130 extant families that exist worldwide (Kirk-Spriggs and Marshall 2017). The list of the top ten most well-represented families in the region, each with over 500 described species, includes Aslidae (1,685), Bombyliidae (1,384), Chironomidae (604), Culcidae (780), Dolichopodidae (770), Limoniidae and Tipulidae (approximately 1,045), Muscidae (1,035), Syrphidae (about 600), Tabanidae (around 800), Tachinidae (1,126), and Tephritidae (approximately 1,000). Among the vast array of species, only a select few are classified as "filth flies" because of their close affiliation with animal excrement, decomposing food, or carrion (Machtinger et al. 2021), hence the focus of the present review.

Some of these flies, such as the house fly, stable fly, face fly (*Musca autumnalis De Geer*), horn fly (*Haematobia irritans*), and little or lesser house fly (*Fannia canicularis*) pose significant concerns for animal production. These filth flies are often closely associated with animals and their excreta, which they preferentially or completely utilise for developing larvae. Adult flies of these species also obtain their nourishment from animals, such as blood, exudates, or other bodily secretions (Machtinger et al. 2021). These flies are not only a nuisance but carriers of disease-causing organisms that can affect both humans and livestock (Geden et al. 2021; Nisar et al. 2021). Furthermore, filth flies play a critical role in the transmission of pathogens such as fungi, bacteria, and viruses that can cause illness (Machtinger et al. 2021). The influence of fly species’ distribution is intricate and remains an enigma despite extensive scientific investigation. The precise causes that restrain fly species from expanding their range are yet to be established. Considering that the distribution of their hosts does not confine most fly species, it is deducible that climate serves as the predominant inhibitor for the growth of fly species ranges (Goulson et al. 2005; Gilles et al. 2008).

### Economic and ecological impact of fly infestation in cattle production

The parasitic relationship that exists between cattle and flies leads to significant financial losses experienced in beef operations (Ling et al. 2020; Warner et al. 2022). For instance, flies decrease feed intake and feed conversion efficacy (FCE) and increase energy demand (Mohammed et al. 2016). This subsequently reduces weight gain and milk production in cattle (Durunna and Lardner 2021; Makhahlela et al. 2022). These effects collectively diminish the profitability of cattle production, with estimates suggesting that the economic threshold of stable fly infestation in cattle is approximately 15 flies per animal (Rochon et al. 2021). However, horn flies generally require intervention when their fly count reach around 200 flies per animal (Brewer et al. 2021). Additionally, the financial losses extend beyond direct production impacts. For instance, the horn fly, which feeds between 24–38 times a day and draws 11–21 mg of blood per feeding (Parra et al. 2013), not only present itself as an annoyance and irritant to the host (Mohammed et al. 2016), but also leads to increased costs for fly control measures and veterinary care to manage the health issues caused by these pests.

Ecologically, fly infestations compromise animal welfare and pose health risks to both humans and animals. Flies serve as vectors for the transmission of contagious diseases and contribute to the prevalence of conditions such as myiasis and mastitis in animals (Mohammed et al. 2016; Makhahlela et al. 2022). Stable flies have been implicated in the transmission of lumpy skin disease (Rochon et al. 2021). The overall economic losses due to reduced productivity and increased management costs, coupled with the ecological impacts on animal welfare and health, can be substantial, further exacerbating the economic burden on cattle producers.

### Conventional and contemporary fly control methods and their limitations

Over a century of research has led to the development and refinement of numerous methodologies to control pest flies, particularly to protect cattle and minimise production losses, employing a variety of chemical, biological, physical, and cultural methods (Cook 2020). Such methods include back rubbers, spray-on, pour-on, insecticide-impregnated ear tags, larvicide oral treatment, biological control, and vacuum fly traps have also been used as methods to control livestock flies (Kienitz et al. 2018; Brewer et al. 2021). However, insecticides act as a temporary management tool, since reliance on their use and long-term efficacy has proven inadequate (Ling et al. 2020). Hence, effectively controlling pests is necessary to increase profitability through improved animal health and performance (Durunna and Lardner 2021).

Botanical repellents sticky traps are frequently used by organic dairy farmers to control fly infestations; however, these methods often have limited effectiveness (Sorge et al. 2015). This limitation is partly due to the rapid reproduction and spread of flies, for instance, horn flies, which can quickly migrate across neighbouring pastures. The productivity of a herd is determined by the difference between the speed at which traps can eradicate and eliminate flies from cattle and the rate at which they naturally emerge in the pastures (Kaufman et al. 2005). A proficient IPM program for house flies and stable flies on dairy farms involves regular removal of bedding, judicious choice of bedding material, utilization of less-toxic insecticides, release and preservation of biological control agents, and deployment of physical controls like traps (Geden and Hogsette 1994; Kaufman et al. 2005). There is currently limited information available on the use of botanical pesticides in conjunction with integrated pest management (IPM) to combat pest flies in cattle production. Therefore, it is crucial to increase awareness of the potential benefits of incorporating botanical pesticides into IPM programs for fly control in cattle production. By promoting the integration of botanical pesticides into IPM programs and raising awareness of their potential, livestock producers can effectively manage fly populations while minimizing the negative environmental and health impacts associated with the use of synthetic pesticides.

#### Fly resistance to existing conventional insecticides

Over 100 years ago, Melander (1914) documented the first case of insecticide resistance, specifically in scale insects that displayed resistance to an inorganic insecticide. Eleven more cases of resistance to inorganic insecticides were reported thereafter. Insecticide resistance occurs when an insect population becomes less sensitive to an insecticide, causing the insecticide to fail to control the insects despite proper product storage, application, and normal environmental conditions (‘Insecticide Resistance Action Committee, IRAC’,; Siddiqui et al. 2023). The use of organic insecticides, such as Dichlorodiphenyltrichloroethane (DDT) was adopted to mitigate the issue of insecticide resistance (Carvalho 2006). However, housefly resistance to DDT was documented by the year 1947. The emergence of resistance to insecticides has been a recurring phenomenon with the introduction of each new class of insecticides, with the timeframe ranging from 2 to 20 years. Insecticide resistance is most likely attributed to the overapplication of synthetic chemicals (Siddiqui et al. 2023). Insects develop insecticide resistance through a variety of mechanisms, the most important of which are behavioural resistance, fitness cost, reduced penetration, target resistance, and metabolic resistance (Siddiqui et al. 2023).

Insects may reduce their exposure to pesticides by changing their behaviours, such as avoiding the toxin or ceasing feeding upon encountering certain insecticides (Khan et al. 2020; Siddiqui et al. 2023; Gul et al. 2023). Resistant insects may absorb the toxin more slowly or develop outer cuticles that slow the penetration of the insecticide (IRAC). The specific binding site of an insecticide in the insect may be genetically modified to prevent the insecticide from binding, rendering the target site incompatible (Khan et al. 2020). Resistant insects may detoxify or destroy the toxin faster or rapidly eliminate it from their bodies. They may possess higher levels or more efficient forms of enzymes that break down insecticides, and these enzyme systems may have broad activity (IRAC). These mechanisms can occur independently or in combination, making effective control of insect populations challenging.

#### The use of odour-based fly repellents

Flies use a combination of odour and visual cues for host location, with odour playing a significant role in attracting them from a distance and the latter become crucial for the landing (Borst and Heisenberg 1982). Through exploitation of this host-seeking behaviour, researchers have created colourful traps and targets, incorporating host attractants, to combat the issue of fly abundance. As part of the IPM program, studies have explored the use of odour-repellents from non-preferred hosts such as zebra and waterbuck skins as alternative approach to control pest fly abundance in livestock (Saini et al. 2017; Olaide et al. 2019; Ogolla et al. 2023). Mireji et al. (2022) recently reviewed odour-based control strategies for tsetse flies in Africa, concluding that artificial bait technologies leveraging long-range olfactory responses to host cues and synthetic blends have effectively reduced certain tsetse fly populations using repellent-odour-based "push" tactics.

Garlic and its bioactive compounds, particularly allicin (diallyl-thiosulfinate), have been shown to possess insect-repellent properties, making them a promising botanical solution for managing pest infestations in livestock (Mohammed et al. 2016; Plata-Rueda et al. 2017; Hagg et al. 2024). The repellent properties of garlic are linked to its sulfur-containing compounds, such as allicin, ajoene, and allyl mercaptan, which are released through the skin and breath after ingestion (Rahman 2007; Zeng et al. 2017). These odorous compounds disrupt fly host-seeking behaviour, potentially reducing fly burden on cattle.

Although several studies have reported garlic’s effectiveness in repelling flies, inconsistencies in fly reduction percentages have been observed. Mohammed et al. (2016) and Durunna and Lardner (2021) found a notable reductions in fly abundance and defensive behaviours in cattle treated with a garlic-based pour-on and those supplemented with garlic, respectively. However, Durunna and Lardner (2021) reported inconsistencies in their second-year findings, where garlic supplementation did not significantly reduce fly numbers. These discrepancies may stem from genetic differences in cattle, environmental conditions, variations in garlic processing methods and inconsistencies in the bioactive sulfur compounds responsible for fly repellence. Factors such as dosage, delivery methods (e.g., feed inclusion vs. topical application), and interactions with other dietary components may also influence garlic’s effectiveness. Future research should focus on quantifying these compounds and optimising supplementation protocols for consistency.

## Historic use of garlic in livestock production

Garlic has been used for centuries across various civilisations for its medicinal and performance-enhancing properties (Rivlin 2001; Rahman 2007). In livestock production, garlic and its bioactive compound, allicin, have been explored for their potential to improve animal health, performance, and product quality. Studies have demonstrated that garlic supplementation in animal diets enhances meat quality, growth performance, and immune response. For example, garlic powder improved meat marbling, firmness in pigs (Chen et al. 2008) and enhanced rumen fermentation and health status in lambs infected with gastrointestinal nematodes (Zhong et al. 2019). Given its reported bioactivity, further in vivo trials are essential to validate garlic’s efficacy in controlling flies and improving livestock production (Ding et al. 2023).

### Chemical profiles of garlic and its by-products

**Fig. 2:**
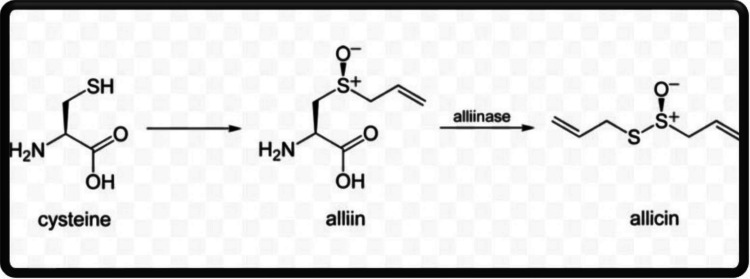
Chemical structure of garlic. Adopted from Abd El-Ghany (2024)

**Fig. 3:**
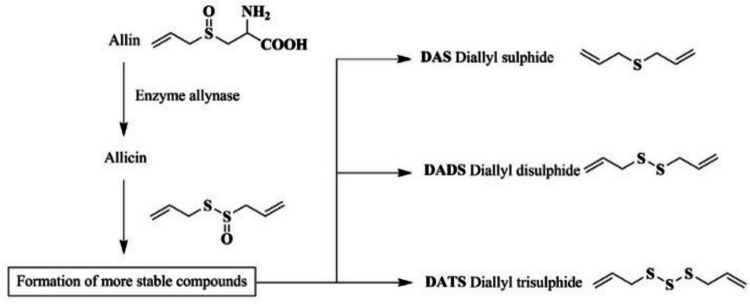
Alliin transformation to more stable compounds. Adapted from Morales-González et al. (2019)

Garlic (Fig. 2) contains a diverse array of bioactive compounds with potential benefits in livestock production (Bose et al. 2014). Key sulfur-based compounds such as allicin, diallyl sulfide (DAS), diallyl disulfide (DADS), and diallyl trisulfide (DATS) are responsible for its insecticidal, antimicrobial, and immune-modulatory properties (El-Saber Batiha et al. 2020; Hardiansyah et al. 2020). Allicin, (Fig. 3) which is enzymatically produced by allinase from alliin when garlic is crushed (Rahman 2007; Chen et al. 2021), is highly unstable and rapidly degrades into more stable sulfur-containing metabolites such as DAS, DADS, DATS, and/or sulfur dioxide (Melguizo-Rodríguez et al. 2022). These stable sulfur compounds contribute to the overall bioactivity and therapeutic effects of garlic.

Despite the instability of allicin, these compounds retain their efficacy in promoting health and combating diseases (Reinhart et al. 2009; Hardiansyah et al. 2020), owing to the presence of these stable sulfur compounds, although their relative proportions may change over time. For instance, garlic essential oil has been found to have insecticidal activity against various pests, including the mealworm beetle and grain moth (Zhao et al. 2013; Plata-Rueda et al. 2017). It is crucial to understand the chemical profiles and stability of sulfur-based compounds in garlic products to optimize their therapeutic potential and ensure consistent quality in both pesticidal, medicinal and culinary applications.

### Garlic as a feed additive in cattle

Garlic has been widely explored as a feed supplement (Table 2) due to its potential to enhance animal health and productivity (Durunna and Lardner 2021). Various forms of garlic, including dried flakes, essential oils, and powders, have been studied for their effects on rumen fermentation, nutrient utilisation, and immune function (Ikyume et al. 2017; Saastamoinen et al. 2019; Durunna and Lardner 2021). These garlic products can be administered to animals via feed inclusion, mineral and salt supplement. Martin and Chaudhry (2024) used a meta-analysis approach to evaluate the impact of various garlic-based feed additives on ruminal fermentability and ruminant performance. Their findings suggested that garlic supplementation positively influences volatile fatty acid production, microbial activity, and overall feed efficiency, further supporting its potential as a functional feed ingredient.

**Table 2:** Studies exploring the use of garlic in ruminant livestock

References	Species studies	Inclusion level	Measurement	Effect
(Mohammed et al. 2016)	Cattle	500 g chopped garlic clove / 200 mL paraffin oil	Flies’ attack rate and animal’s defensive behaviour	Reduced fly count
(Hasan et al. 2015)	Goat	25 mL and 50 mL of 10% water solution of garlic	Egg per gram (EPG) count for gastrointestinal parasites, live weight, and hematological parameters	Decreased EPG count, increased weight gain, significant changes in the hematological parameters
(Zhong et al. 2019)	Sheep	50 g/kg garlic powder	Growth performance, rumen fermentation, and the health status of lambs	Increased lambs’ average daily gain, digestibility of dry matter, and crude protein
(Rabee et al. 2025)	Sheep	2% of garlic powder	Growth performance, blood metabolites, immunity, rumen fermentation, and bacteria community in lambs	Improved the performance and immune status of growing lambs
(Xu et al. 2024)	Sheep	80 g/kg dry matter garlic skin	Growth performance, rumen and fecal microbiota, serum and urine metabolism, and transcriptomics of rumen epithelial cells in fattening sheep	Improved the energy metabolism and immune function of fattening sheep

In cattle, garlic supplementation has shown promising results in improving feed conversion efficiency (FCE), weight gain, and fly repellence (Mohammed et al. 2016; Rossi et al. 2018; Durunna and Lardner 2021). However, the precise metabolic pathways through which garlic exerts its fly-repellent effects remain unclear, requiring further research to determine optimal inclusion levels, delivery methods, and long-term benefits in cattle production. To effectively implement garlic supplementation for fly control in cattle, it is essential to establish standardised protocols regarding dosage, formulation, and delivery methods. Optimal dosages should be determined to ensure effective fly repellence while maintaining feed palatability and avoiding potential negative effects on animal health. Previous studies suggest that inclusion levels ranging from 0.2% to 8% of the diet can improve growth performance and immune function as shwon in Table 2. However, fly repellence-specific dosages require further evaluation. Addressing these factors would provide necessary information to cattle farmers for practical application and provide a viable alternative to synthetic pesticides.

### Garlic implications on metabolic pathways

Once ingested, allicin undergoes rapid metabolism in the gastrointestinal tract, converting into stable sulfur metabolites such as allyl mercaptan and S-allyl mercapto cysteine (SAMC), which may modulate immune and metabolic functions (Rahman 2007; Ansary et al. 2020). Some studies suggest that garlic-derived metabolites can influence red blood cell permeability, immune responses, and antioxidant activity (Miron et al. 2000; Rao et al. 2015; Mi ekus et al. 2020), potentially contributing to fly resistance in cattle. However, given that allicin is short-lived and rapidly transforms into more stable compounds such as DAS, DADS and DATS (Rao et al. 2015; Mi ekus et al. 2020), future studies should focus on nutrigenomic evaluations to determine how garlic supplementation regulates immune-related and insect-repellent pathways in cattle, which could provide valuable insights for sustainable pest control strategies.

## Potential immune-mediated fly control mechanisms of garlic in cattle

Flies have devolved sophisticated mechanisms to evade and suppress the host's immune system, ensuring their survival and completion of their life cycle (Vilcinskas 2013). These mechanisms include molecular mimicry, immune suppression by regulatory T cells, antigenic camouflage, and modulation of host immunological factors (Caljon et al. 2006; Bezie 2014). For instance, tsetse fly saliva contains Gloss2, an immunoregulatory peptide that inhibits the secretion of key pro-inflammatory cytokines such as TNF, IFN-γ, and IL-6, impairing the host’s ability to mount an effective immune response (Lackie 1988; Caljon et al. 2006). This suppression allows flies to evade early detection and elimination, thereby enhancing their chances of survival and increasing the disease transmission.

The primary immune response to fly bites occurs at the skin, involving the activation of immune cells such as macrophages, dendritic cells, monocytes, and lymphocytes, as well as the production of antibodies and cellular immune responses (Gomes and Oliveira 2012; Abdeladhim et al. 2014). Rapid immune activation at the bite site is often associated with a higher level of resistance to flies (Naessens et al. 2003). However, flies’ ability to supress immune responses compromises this defense mechanism. Given this, strategies that enhance cattle’s natural immune response could provide a sustainable means of fly control.

Garlic has been identified as a natural immunomodulator with potential in improving fly resistance in cattle (Durunna and Lardner 2021). Although there is there is limited literature specifically addressing garlic’s influence on immune responses towards fly repellence, its effects on enhancing immune function suggest a promising role (Arreola et al. 2015). Studies have shown that garlic compounds, particularly S-allyl cysteine, enhance antioxidant enzyme activity and stimulates the immune response of ruminants by increasing the levels of immunoglobulins, such as immunoglobulin A, G, and immunoglobulin M (Kekana et al. 2020; Redoy et al. 2020; Kewan et al. 2021). Furthermore, garlic supplementation has been linked to increases activation of immune cell types, including macrophages, lymphocytes, natural killer cells, dendritic cells, and eosinophils, through mechanisms such as modulation of cytokine secretion, immunoglobulin production, phagocytosis, macrophage activation, cellular co-receptor expression, class switching, lymphocyte expression, and histamine release (Mahima et al. 2012; Arreola et al. 2015; Melguizo-Rodríguez et al. 2022). These immunomodulatory effects suggest that garlic could enhance cattle’s ability to mount a stronger immune response against fly bites, potentially reducing fly burden and mitigating disease transmission. Moreover, diet-driven immune responses have been shown to influence gene expression, affecting an animal’s resilience to environmental stressors, including parasite infestations (Le Coz et al. 2021; Brouklogiannis et al. 2023). While research on garlic’s direct impact on immune-mediated fly resistance in cattle is still limited, its effects on immune function and inflammation modulation warrant further investigation.

## Genetic basis of fly resistance in cattle

Fly resistance in livestock, particularly in cattle, can be influenced by various factors, including genetic traits and gene expression. Studies have shown that some cattle are naturally resistant to flies, and both nutrition and genetic factors influence this resistance. For instance, genetic analyses have demonstrated significant genetic variation in fly resistance traits in cattle, with heritability estimates ranging from 10 to 80% (Brown et al. 1992; Basiel et al. 2021). This resistance can be observed as the difference in the number of flies on some animals compared to their herd-mates and is influenced by factors such as breed, size of the animals, and individual differences (Brown et al. 1992). There are differences among breeds of cattle in resistance to parasites, with Zebu (*Bos indicus*) cattle breeds being most adaptable and acquiring fly resistance relatively faster than the European (*Bos taurus*) and some African indigenous (*Sanga*) cattle breeds (Mattioli et al. 2000; Steelman et al. 2003; Mwai et al. 2015).

The *Bos indicus* breeds of cattle, domesticated in harsh environmental conditions, are believed to have developed superior natural resistance to external stressors and parasites (Machado et al. 2010). Studies have shown that Zebu cattle exhibit better resistance to various pests and diseases, including trypanosomiasis transmitted by the tsetse fly (Mwai et al. 2015; Pal and Chakravarty 2020). Selective breeding for fly resistance has been proposed as a sustainable management method to control fly infestation (Steelman et al. 1993; Oyarzún et al. 2008). This suggests that the genetic foundation for fly resistance in cattle, particularly *Bos indicus* breeds, can be leveraged to enhance their ability to withstand fly infestations and other environmental stressors. Individual variation within and across breeds of beef cattle in resistance to horn flies has also been observed, with some cattle consistently hosting fewer horn flies, indicating a genetic basis for fly resistance (Steelman et al. 1993; Guglielmone et al. 2000). Identifying the mechanisms by which resistant cattle prevent high fly infestation is an essential step for the development of predictive markers for resistance. Although some work on the heritability and dynamics of host resistance, and the factors that can influence it has been undertaken, the mechanisms by which the resistant host prevents the pest fly infestation on an individual remain to be elucidated.

Gene expression, the essential biological process through which information encoded within a gene is transcribed into functional gene products, including proteins and non-coding RNAs, plays a crucial role in livestock production. The expression of genes in livestock is influenced by various factors, including genetic variation, environmental conditions including nutrients, bioactive compounds, and management practices (Berry et al. 2011; Mierziak et al. 2021). For example, certain genes may be expressed more or less strongly in response to changes in diet, temperature. Weyrich et al. (2019) investigated the transmission of DNA methylation changes to male offspring following paternal exposure to either a diet with reduced protein content or an increase in temperature. Through selective breeding, gene expression can be modified to enhance desirable traits in livestock (Kiplagat et al. 2012). Equally important, phytogenic diets can also stimulate expression of genes (Mierziak et al. 2021) involved in fly resistance.

## Potential nutrigenomic effects of garlic supplementation on fly repellence in cattle

In molecular animal nutrition, a subset of the broader field of nutrigenomics addresses the genetic basis of response to diet and, in parallel, the variations in dietary responsiveness among animals that are assignable to genotype (German et al. 2011). Utilizing different “omics” techniques from a variety of disciplines, (such as genomics, epigenomics, transcriptomics, proteomics, and metabolomics), nutrigenomics in practice examines animal cellular and molecular responses to different dietary nutrients, revealing the global influence of nutrients and phytochemicals on animal genomes, methylomes/epigenomes, transcriptomes, proteomes, and metabolomes, respectively (García-Cañas et al. 2010; Hasan et al. 2019; Haq et al. 2022). Transcriptomics has been instrumental in identifying genes associated with feed efficiency and metabolism in ruminants (Zhang et al. 2019; Lam et al. 2020; Lindholm-Perry et al. 2022). For instance, RNA-Seq technology has been used to identify functional candidate single nucleotide polymorphisms (SNPs) within genes associated with feed efficiency and metabolic pathways in cattle breeds (Higgins et al. 2019; Lam et al. 2020).

The influence of dietary bioactive compounds on gene expression regulation is significant in nutrigenetics and nutrigenomics. Dietary bioactive compounds, such as polyphenols, vitamins, flavonoids, carotenoids, glucosinolates, isothiocyanates, terpenes, fatty acids, allicin, DAS, DADS, DATS, S-allycystine, and S-allylmercaptocysteine, transfer information from the external environment and influence gene expression (Takemura et al. 2013; Sheoran et al. 2017). Garlic contains most of these bioactive compounds, which can influence the expression of genes related to immune function and metabolism. Although there is lack of nutrigenomic evaluation of garlic in cattle, studies done on non-ruminants suggest that garlic can upregulate genes related to immunity, apoptosis, and xenobiotic metabolism (Charron et al. 2015; Sheoran et al. 2017). For instance, Sheoran et al. (2017) reported that garlic powder significantly enhanced the relative mRNA expression of toll-like receptor cell markers, indicating its potential to stimulate the immune responses in livestock. Recent studies, (Xu et al. 2024; Rabee et al. 2025) found that garlic supplementation influences gut microbiota composition and metabolic pathways, potentially affecting immune regulation in ruminants. Additionally, garlic extracts activate the nuclear factor erythrobia-2 related factor 2-antioxidant response element pathway, enhancing expression of protective enzymes such as heme oxygenase-1 and glutamate-cysteine ligase modifier subunit (Mierziak et al. 2021).

Despite thsese findings, there is a significant gap in the literature regarding studies that directly investigated garlic’s effects on gene expression related to fly resistance in cattle. While garlic’s potential to influence immune function and metabolism is well documented (Melguizo-Rodríguez et al. 2022; Xu et al. 2024; Rabee et al. 2025), little is known about how these changes could affect fly behaviour, feeding patterns, or reproduction. This knowledge gap highlights the importance for further research to assess garlic’s nutrigenomic role in fly repellence, specifically in cattle. Future studies should use RNA-seq to identify differentially expressed genes associated with immune response and oxidative stress, and skin barrier function that may contribute to fly repellence. Futhermore, epigenetic studies may uncover regulatory modifications (such as DNA methylation or histone modifications) that influence gene expression related to fly resistance. Additionally genome-wide association studies (GWAS) could help identify genetic markers linked to fly repellence and assess the impact of garlic supplementation on these traits. Understanding the genetic basis of fly resistance and leveraging nutrigenomic strategies may enhance resilience, reducing reliance on synthetic pesticides while improving productivity and animal welfare.

## Conclusions

Garlic supplementation in livestock production presents a promising natural strategy for fly repellence, enhanced animal health, and improved meat production. Additionally, selecting fly-resistant cattle and integrating garlic-based phytogenic diets could offer a sustainable and environmentally friendly approach to mitigating fly infestations. The synergistic effects of genetic resistance and dietary interventions hold significant potential for reducing reliance on synthetic pesticides, and subsequent improvement of cattle productivity and welfare. As such, further research is important to determine the precise nutrigenomic mechanisms through which garlic influences fly resistance. This integrated approach could enhance resilience in intensive production systems, contributing to more sustainable and profitable livestock farming.

## Data Availability

Not applicable.
